# Localization and density of phoretic deutonymphs of the mite *Uropoda orbicularis* (Parasitiformes: Mesostigmata) on *Aphodius* beetles (Aphodiidae) affect pedicel length

**DOI:** 10.1007/s00114-014-1150-x

**Published:** 2014-02-07

**Authors:** Daria Bajerlein, Wojciech Witaliński

**Affiliations:** 1 Department of Animal Taxonomy and Ecology, Faculty of Biology, Adam Mickiewicz University, Umultowska 89, 61-614 Poznań, Poland; 2 Department of Comparative Anatomy, Institute of Zoology, Jagiellonian University, Gronostajowa 9, 30-387 Kraków, Poland

**Keywords:** Pedicel, Phoresy, Attachment structures, Uropodina, Mites, Dung beetles

## Abstract

The phoretic stage of Uropodina mites is a deutonymph with developed morphological adaptations for dispersal by insects. Phoretic deutonymphs are able to produce a pedicel, a stalk-like temporary attachment structure that connects the mite with the carrier. The aim of our study was to determine whether localization and density of phoretic deutonymphs on the carrier affect pedicel length. The study was conducted on a common phoretic mite—*Uropoda orbicularis* (Uropodina) and two aphodiid beetles—*Aphodius prodromus* and *Aphodius distinctus*. Our results show that pedicel length is influenced by the localization of deutonymphs on the body of the carrier. The longest pedicels are produced by deutonymphs attached to the upper part of elytra, whereas deutonymphs attached to femora and trochanters of the third pair of legs and the apex of elytra construct the shortest pedicels. In general, deutonymphs attached to more exposed parts of the carrier produce longer pedicels, whereas shorter pedicels are produced when deutonymphs are fixed to non-exposed parts of the carrier. A second factor influencing pedicel length is the density of attached deutonymphs. Mean pedicel length and deutonymph densities were highly correlated: higher deutonymph density leads to the formation of longer pedicels. The cause for this correlation is discussed, and we conclude that pedicel length variability can increase successful dispersal.

## Introduction

Many animals spend their entire or some of their life cycle attached to other organisms or substrates. This life strategy is common in ectoparasites, epizoic species, sedentary benthic organisms, and inhabitants of wave-swept shores. It also requires adaptations that enable attachment. A wide variety of attachment mechanisms are found in invertebrates such as monogeneans (Platyhelminthes), gastropods and bivalves (Mollusca), barnacles (Crustacea), beetles (Insecta), spiders and mites (Arachnida), and sea urchins (Echinodermata). Attachment structures vary in structure, localization, and origin, and their classic examples are suckers, food pads, hooks, claws, spines, and clamps. The ability for permanent or temporary attachment may also be enabled by gland(s) producing a sticky substance that is released outside of the body. Such an attachment mechanism is common and well known in monogeneans, mussel byssus, spiders, barnacles, and sea urchins (Whittington et al. 2004; Bromley and Heinberg 2006; Brazee and Carrington 2006; Aldred and Clare 2008; Santos et al. 2009; Dodou et al. 2011; Farsad and Sone 2012; Sahni et al. 2012; Santos and Flammang 2012). A sticky substance known as a mussel byssus thread occurs in bivalves and enables adhesion to underwater substrates. Spiders produce a sticky substance that has a variety of applications and may be used not only for attachment but also for dispersal, procuring food, and cocoon production. A highly specialized structure used only for temporary attachment to the carrier, which recently has been examined (Bajerlein and Witaliński 2012; Bajerlein et al. 2013), is an anal pedicel produced by phoretic deutonymphs of Uropodina mites.

Phoresy as a way of dispersal is common in mites inhabiting unstable and patchily distributed microhabitats such as animal dung, compost soil, social hymenopteran insect (e.g., bumblebees) and bird nests, and carrion (Faasch 1967; Evans 1992; Athias-Binche 1994; Mašán 2001; Bajerlein et al. 2006; Krantz and Walter 2009; Bajerlein 2011). If the microhabitat is ephemeral or its conditions become unfavorable, deutonymphs of Uropodina disperse by using coexisting arthropods in order to colonize a new habitat, more suitable for further development. Phoretic deutonymphs of Uropodina mites use primarily insects for dispersal and among these mostly beetles from the families Aphodiidae, Brenthidae, Cerambycidae, Geotrupidae, Histeridae, Passalidae, Scarabaeidae, Silphidae, and Staphylinidae (Athias-Binche et al. 1993; Wiśniewski and Hirschmann 1993; Mašán 2001; Bajerlein 2011). Phoretic relationships between deutonymphs of Uropodina and beetles date back to the Eocene (Dunlop et al. 2013). In most Uropodina, the pedicel resembles a straight stalk of variable length with enlarged termini (Faasch 1967; Bajerlein and Witaliński 2012; Bajerlein et al. 2013). One terminus of the pedicel adheres to the deutonymph’s anus, and the second terminus is attached to the surface of the carrier. From the biomechanical point of view, the termini of the pedicel constitute an example of a mushroom shaped attachment structures. Such adhesive biological structures are common and known, e.g., in molluscs (mussel byssus threads), spiders (dragline attachment), echinoderms (tube feet), and Cnidaria (polyp foot), and were studied in detail by various authors (Gorb and Varenberg 2007; Gorb et al. 2007; Carbone et al. 2011). The substance for the pedicel is produced by the pedicellar gland occurring dorsally over the colon in the rear part of the deutonymph’s body and is secreted outside through the anus (Bajerlein and Witaliński 2012). The formation of a pedicel occurs when a phoretic deutonymph situates itself with its anus on the carrier, secretes the pedicellar substance, and gradually straightens its fourth pair of legs. Longer pedicels are formed as a result of elongation of one that has already been produced, i.e., a deutonymph attached to the carrier via a pedicel starts to walk forward and extends the latter. After colonizing a new habitat, the deutonymph detaches from its carrier and the pedicel usually remains on the carrier’s body (Faasch 1967). The pedicel is a temporary structure, and a single deutonymph may produce many pedicels throughout life. Phoretic deutonymphs of Uropodina may attach to various parts of the carrier, and topical specificity was found in deutonymphs of some species (Schwarz et al. 1998; Bajerlein and Błoszyk 2004; Błoszyk et al. 2006).

Although pedicel production is common in Uropodina, literature on this subject is scarce. Phoretic deutonymphs attached to their host were already noticed and illustrated in the eighteenth century (De Geer 1768), but the first detailed observations were presented by Faasch (1967), who analyzed adaptation to phoresy and phoretic behavior in *Uropoda orbicularis* (Müller, 1776) and *Uroobovella marginata* (C. L. Koch, 1839). Recent studies conducted by Bajerlein and Witaliński (2012) and Bajerlein et al. (2013) described the anatomy and fine structure of the pedicellar gland as well as morphological diversity of pedicels by transmission (TEM) and scanning electron microscopy (SEM), but also revealed that pedicels within the same species are highly variable in length. Vitzthum (1943) noted that pedicels may be short or long. In the laboratory, more detailed observations were conducted by Faasch (1967) who found that in the absence of food, pedicels of *U. marginata* become shorter and eventually cease to be produced. Moreover, in *U. orbicularis*, the frequency of pedicel production is negatively correlated with pedicel length. According to Faasch (1967), pedicels in *U. marginata* are characterized by variable length, whereas in *U. orbicularis*, the length is relatively constant. Since the latter observation is in contrast to our previous results (Bajerlein et al. 2013), we carried out ecomorphological studies to determine the factors affecting pedicel length and, in particular, to examine whether deutonymph localization and density on the carrier are important for pedicel length. Thus, two hypotheses were tested as follows: (1) pedicel length differs between different attachment sites on the carrier body, and (2) pedicel length is affected by deutonymph density.

## Materials and methods

The study was conducted on a common phoretic uropodid species *U. orbicularis* and two dung beetle species *Aphodius prodromus* (Brahm, 1790) and *Aphodius distinctus* (O. F. Müller, 1776) (Aphodiidae).

### Collection of beetles and mites

Adults of aphodiid beetles with phoretic deutonymphs of *U. orbicularis* were collected in May and June of 2003–2004 during field studies on a pasture grazed by cattle in the western part of Poland (Wielkopolska region). Beetles and mites were collected using six dung-baited pitfall traps filled with ethylene glycol solution. Every 7 days, insects and mites were gathered and then stored in 75 % ethylene alcohol.

### Study species *Uropoda orbicularis*

is a mite species recorded in Europe and North America (Wiśniewski and Hirschmann 1993; Majka et al. 2007). It is present in unstable microhabitats such as animal dung and compost soil, and phoretic dispersal is a vital life strategy in this species (Bajerlein 2011). *U. orbicularis* is one of the most frequently observed phoretic Uropodina mites. Phoretic deutonymphs are found on various beetles, but most frequently on coprophilous beetles from the families of Aphodiidae, Geotrupidae, Scarabaeidae, Histeridae, and Hydrophilidae (Wiśniewski and Hirschmann 1993; Bajerlein 2011). Phoresy of *U. orbicularis* on coprophilous beetles including the two studied *Aphodius* species was observed in Poland in spring and autumn (Bajerlein 2011). *A. prodromus* and *A. distinctus* are beetles of the family Aphodiidae and dominate dung beetle communities in Poland in early spring and in autumn. Both species are saprophagous and are often found in dung of herbivorous mammals, compost and in decaying plants (Stebnicka 1976).

### Deutonymph localization on the carrier and pedicel length

**Fig. 1:**
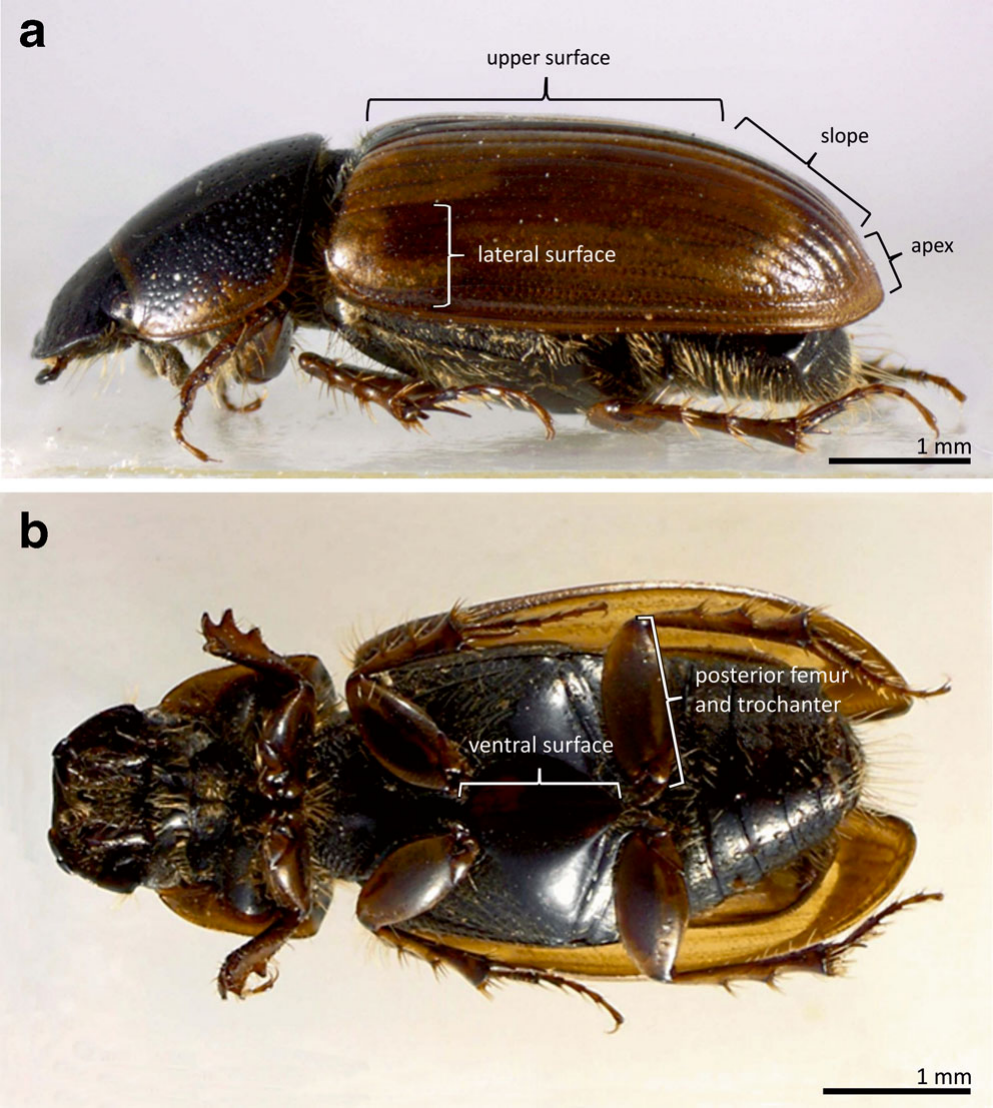
Lateral (**a**) and ventral (**b**) view of the body of *Aphodius prodromus* with indication of the analyzed body parts

In order to determine whether the site of deutonymph attachment affects pedicel length, we compared pedicel length between deutonymphs attached to the following six body parts of *A. prodromus*: (1) area formed by femora and trochanters of the third pair of legs; (2) ventral surface between second and third pair of legs; and (3) apex, (4) slope, (5) upper part, and (6) lateral surfaces of elytra (Fig. 1a, b). The elytra were divided into four regions for more precise analysis (Fig. 1a). Measurements of 40 pedicels collected from each of the six body parts were made. To exclude the possible influence of deutonymph density on pedicel length, we analyzed cases with one pedicel attached to a given body part of the carrier or cases in which the distance between the site of pedicel attachment of two or more deutonymphs was at least three times larger than deutonymph body length. This distance precludes contact between deutonymphs forming pedicels at the same time.

### Deutonymph density and pedicel length

To determine the relationship between density of phoretic deutonymphs and pedicel length, we compared the length of pedicels attached to the area that is formed by the femur and trochanter of the third pair of legs in *A. prodromus* and *A. distinctus*, one of the most frequently infested body parts of these beetles (Bajerlein and Błoszyk 2004). *U. orbicularis* does not show preferences toward either of these two aphodiid species (Bajerlein and Błoszyk 2004; Bajerlein 2011). We calculated mean pedicel length (MPL) for five categories of deutonymph density (1–5). The first category (1) involved cases when only one deutonymph was attached to the considered area of hind legs. The other categories (2–5) included cases when two, three, four, and five deutonymphs were attached. In fact, this part of the body of the beetle can be infested by more than five deutonymphs, but this is less frequently observed and we excluded such cases from analyses. To examine the relationship between size of phoretic deutonymphs and the size of infested body part, we measured the area of 40 phoretic deutonymphs chosen randomly and the area formed by the femora and trochanters of *A. prodromus* (*N* = 40) and *A. distinctus* (*N* = 40).

### Measurements

All measurements were taken from digital images obtained using an Olympus SZ61 stereomicroscope fitted with a camera and Cell A software (Olympus Corporation, Tokyo, Japan). Material for analysis was prepared as follows. First, a beetle with attached deutonymphs was taken from the alcohol and gently dried using a paper towel. Then, it was placed on a Styrofoam pad covered with double-sided sticky tape. Next, photos of the following beetle body parts were taken: dorsal side (for measurement of beetle length, i.e., distance from the front edge of the pronotum to the posterior end of elytra), and body parts with attached deutonymphs. After taking photographs, pedicel carrier termini were detached from the beetle using a dissection needle. In this way, pedicels were still attached to the deutonymphs. In order to measure the length of the pedicel, such deutonymphs were placed dorsally on a Styrofoam pad with their pedicels parallel to its surface and photographs were taken. The length of the pedicel was routinely measured as the distance between its two termini.

### Statistical analysis

Differences in length between pedicels attached to different body parts of *A. prodromus* were tested using one-way analysis of variance (ANOVA). For the purpose of multiple pairwise comparisons, Tukey’s honest significant difference (HSD) test was used. In order to analyze whether deutonymph density affected pedicel length, first we used one-way ANOVA to evaluate the significance of carrier species (*A. prodromus* and *A. distinctus*) on pedicel length. Although differences in body size between these species were statistically significant (F_(1,78)_ = 189.19, *P* < 0.05), carrier species did not significantly affect pedicel length (F_(1,652)_ = 0.34906, *P* > 0.05). In the next step, we combined deutonymph densities from these species and used one-way ANOVA to test how deutonymph density affected pedicel length. The numbers of analyzed cases within particular categories of deutonymph density were as follows: *N* = 80 for category 1, *N* = 82 for category 2, *N* = 60 for category 3, *N* = 39 for category 4, and *N* = 14 for category 5. For the purpose of multiple pairwise comparisons, Tukey’s HSD test for unequal samples was used. Additionally, Pearson’s correlation coefficient (*r*) was calculated for deutonymph densities and pedicel length. A 5 % level of significance was accepted in all analyses. All analyses were done using Statistica 10 (StatSoft, Inc. 1984–2011).

## Results

**Fig. 2:**
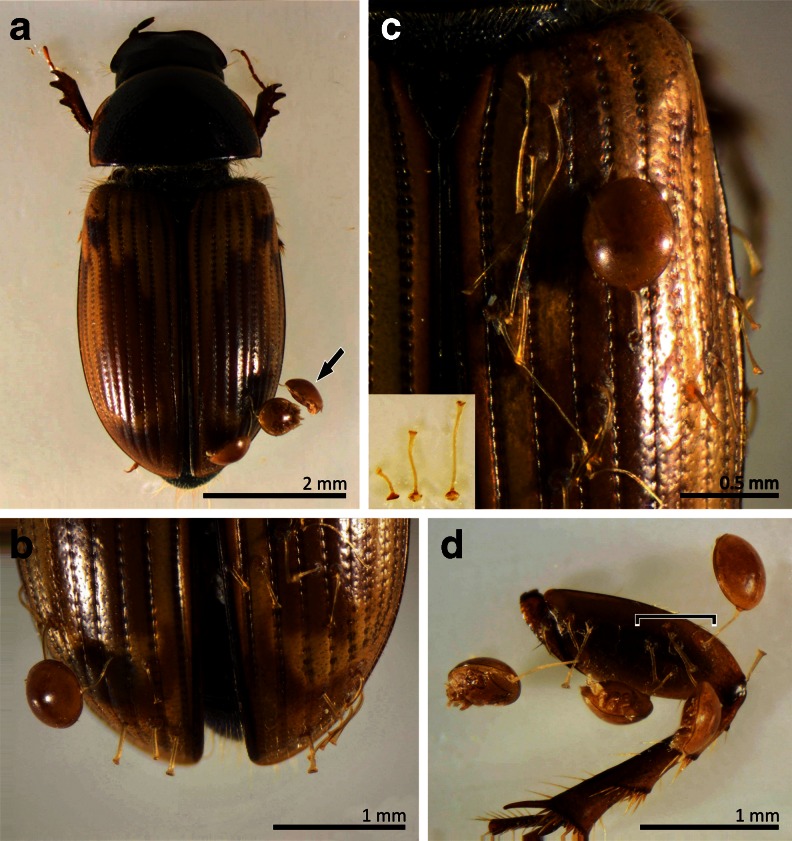
Examples of localization of phoretic deutonymphs of *Uropoda orbicularis* on *Aphodius prodromus*. **a** Two deutonymphs attached to the slope and one (*arrow*) attached marginal to the lateral surface of the elytron. Note that the second elytron is not infested. **b** One deutonymph and a number of pedicels attached to the apex of elytra. A group of pedicels is visible on the upper part of the right elytron. **c** Upper and lateral parts of elytron showing one attached deutonymph and many pedicels of variable length, much longer on the upper part than the lateral part. Note that considerably elongated pedicels are much narrower than shorter ones. Inset: three pedicels collected from apex, slope, and upper part of elytra (from *left* to *right*). Note difference in length. **d** A group of deutonymphs and free pedicels attached to the femur and trochanter of the hind leg. Despite the close location of several pedicellar attachments on the beetle leg (indicated by a *bracket*), the deutonymphs are distant from each other

**Fig. 3:**
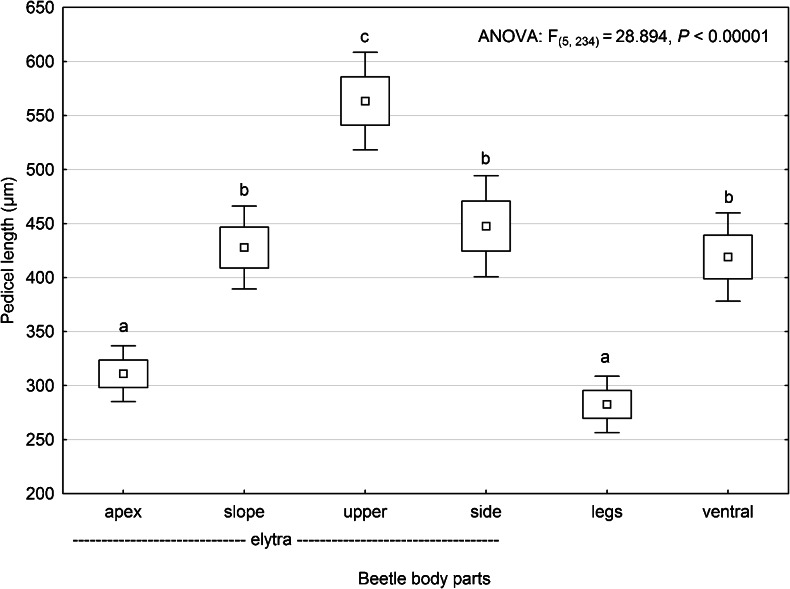
Mean pedicel length in deutonymphs (*N* = 40) attached to different parts of *Aphodius prodromus*. Apex, slope, upper and side—represent apex, slope, upper flat surface and lateral surfaces of elytra, respectively; legs—femora and trochanters of the third pair of legs, and ventral—ventral surface between the second and the third pair of legs. *Boxes and vertical bars* denote standard errors of the means and 0.95 confidence intervals, respectively. Different *letters* denote significant differences between experimental groups in pairwise comparison (*P* < 0.05)

Our study revealed that the localization of phoretic deutonymphs of *U. orbicularis* on adult *A. prodromus* (Fig. 2) affects pedicel length (ANOVA; F_(5,234)_ = 28.894, *P* < 0.05) (Fig. 3). The longest pedicels are produced by phoretic deutonymphs attached to the upper part of elytra: MPL = 563.4 μm (confidence interval (CI): 518.2–608.6) and the shortest by deutonymphs attached to the area formed by femora and trochanters of the third pair of legs: MPL = 282.6 μm (CI: 256.5–308.8) and the apex of elytra: MPL = 311.0 μm (CI: 285.2–336.8) (Fig. 3). MPLs of deutonymphs attached to other parts of the carrier were similar in size: 447.6 μm (CI: 400.7–494.4) for lateral surfaces of elytra, 427.7 μm (CI: 389.4–466.1) for the slope of elytra, and 419.0 μm (CI: 378.1–459.8) for the ventral surface between the second and third pairs of legs of beetles (Fig. 3).

**Fig. 4:**
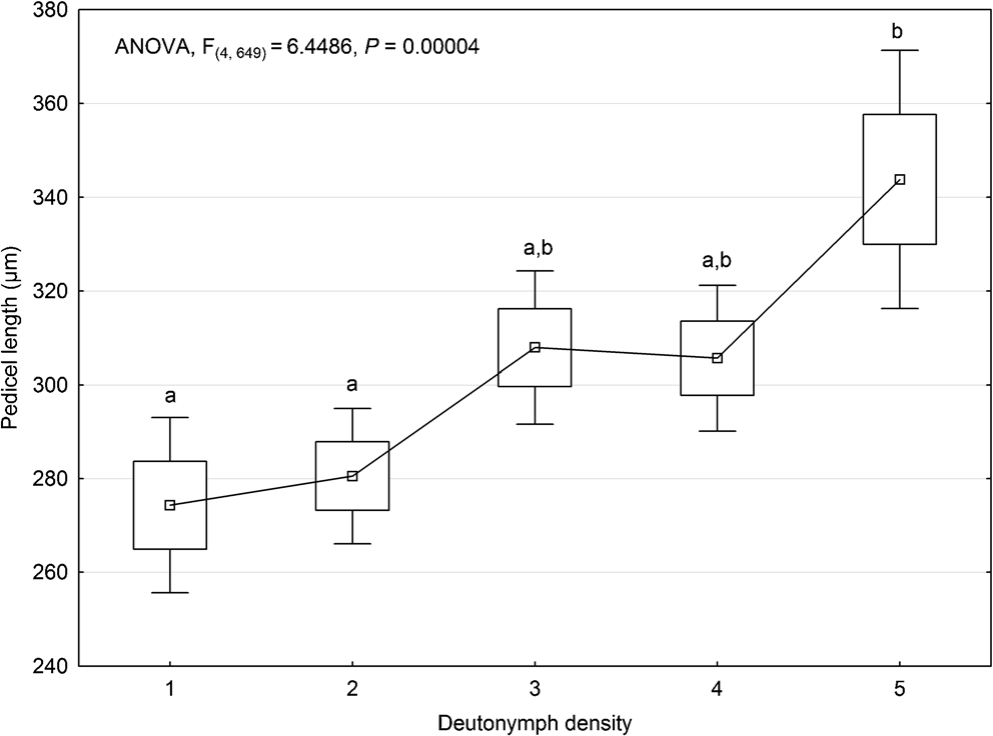
Mean pedicel length of phoretic deutonymphs of *Uropoda orbicularis* found in different deutonymph density categories (number of deutonymphs attached to the femur and trochanter of the third pair of legs). *Boxes and vertical bars* denote standard errors of the means and 0.95 confidence intervals, respectively. Different *letters* denote significant differences between experimental groups in pairwise comparisons (*P* < 0.05)

**Fig. 5:**
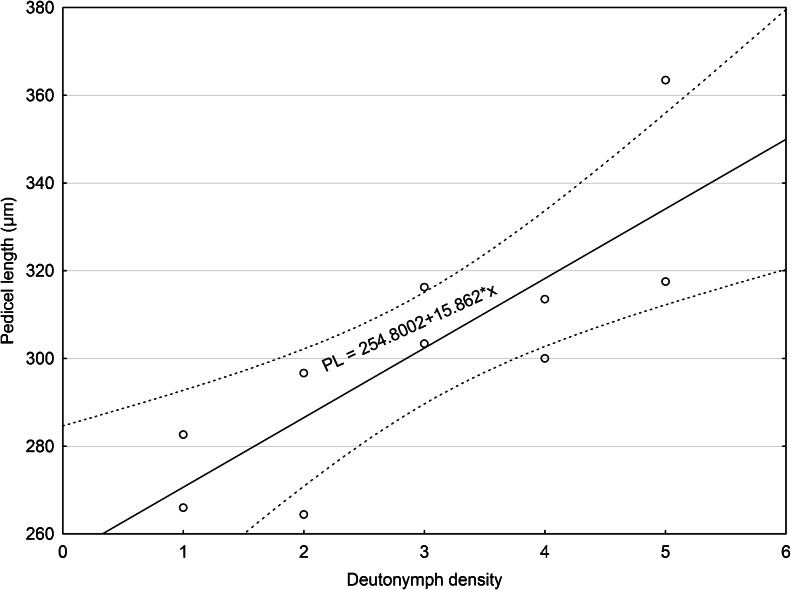
Scatter plot between mean pedicel length in *Uropoda orbicularis* and five categories of deutonymph density (1–5). The categories (1–5) include cases when one, two, three, four, and five deutonymphs were attached. Number of measurements for each category of density is as follows: *N*(1) = 80, *N*(2) = 164, *N*(3) = 180, *N*(4) = 156, *N*(5) = 70

Our analysis did not falsify the hypothesis that increasing density of phoretic deutonymphs of *U. orbicularis* affects pedicel length (ANOVA; F_(4,649)_ = 6.4486, *P* < 0.05) (Fig. 4). We found a strong positive correlation between MPL and deutonymph density *r* = 0.8 (*P* < 0.05) (Fig. 5). The shortest pedicels are formed in cases when only one phoretic deutonymph is attached to the third pair of legs of beetles. The highest MPL was observed when five deutonymphs were attached. In cases of high deutonymph density (≥4), we often observed that the attached mites formed pedicels of variable length (Fig. 2d). One or two deutonymphs have pedicels of standard length, i.e., characteristic for the case of only one attached deutonymph. Other deutonymphs produced longer pedicels, some of which were unusually long (Fig. 2d). The mean areas of the femur and trochanter in *A. prodromus* and *A. distinctus* were 655,141.6 µm^2^ and 465,162.5 μm^2^, respectively. The mean area of the body of phoretic deutonymphs of *U. orbicularis* was 272,971.8 μm^2^. Thus, there are 2.4 and 1.7 tightly packed deutonymphs for each potential attachment site in *A. prodromus* and *A. distinctus*, respectively.

## Discussion

The mechanisms of attachment in Uropodina have rarely been studied; *U. orbicularis* is one of the species that has received more attention (Faasch 1967; Bajerlein and Witaliński 2012; Bajerlein et al. 2013). We first examined the ecomorphology of the pedicel in phoretic deutonymphs of *U. orbicularis*. Previous experiments by Faasch (1967) were focused on biological factors affecting pedicel length in *U. orbicularis* and *U. marginata* such as starvation and the frequency of pedicel formation and shown that under prolonged starvation, phoretic deutonymphs produce shorter pedicels or even cease production altogether. Further, if a deutonymph produces a series of successive pedicels, the subsequent pedicels are shorter. In contrast to Faasch (1967), we examined pedicel length using deutonymphs collected in the field, i.e., in non-starved individuals and analyzed pedicel length in relation to the site of attachment and deutonymph density. We found that pedicel length is influenced by both of these factors.

A study on topical specificity of *U. orbicularis* has shown that phoretic deutonymphs of this species may attach to various parts of a beetle, but some parts are preferred (Bajerlein and Błoszyk 2004). Most frequently phoretic deutonymphs of this species are found on elytra and on the third pair of legs. In general, the number of phoretic deutonymphs is higher on the rear part of the beetle.

We have shown that deutonymphs attached to the legs of the carrier form shorter pedicels in comparison to deutonymphs fixed to the elytra, especially their upper part. In general, deutonymphs form longer pedicels when attached to the exposed parts of the carrier (upper part and lateral surfaces of elytra), and shorter pedicels when attached to non-exposed parts (apex of elytra and third pair of legs). Differences in pedicel length in mites recorded on different parts of the carrier may reflect the risk of being detached. Short pedicels are likely produced when deutonymphs are attached to “safe” body parts, i.e., sites that protect the deutonymph from being dislodged when a beetle digs a tunnel in soil or in dung. In particular, such safe places include the legs and the apex of the elytra. Deutonymphs attached to legs are situated underneath the beetle and are protected by its body. Similarly, deutonymphs that settle on the apex of the elytra are located behind the carrier, so they do not have direct contact with the surrounding soil. A long pedicel allows for greater mobility. Presumably, a mite with a long pedicel can walk on the surface of its carrier and position itself in places with a lower risk of being detached. This explanation seems to be most probable and evidenced by decreasing length of pedicels from upper part of elytra to the apex. Another explanation for the limited length of pedicels is related to the mode of elongation. During pedicel formation, a mite walks forward to extend the pedicel (Faasch 1967), what is possible due to pedicel high expandability, but requires a free surface on which the mite can walk. Probably, this is easier for deutonymphs attached to the upper surface of elytra than to the apex. Nevertheless, this hypothesis seems rather unlikely, since our results have shown that even deutonymphs attached to the femur are able to produce long pedicels and, moreover, the pedicels of subsequently attached deutonymphs can be longer than those previously attached.

The formation of pedicels of various length by deutonymphs attached to the elytra may be associated with flight of the beetle. During flight aphodiid beetles hold the elytra open and long pedicels may facilitate keeping deutonymphs attached, but presently we know nothing about deutonymph behavior during carrier flight.

The adhesive strength of a glue is affected by its chemistry and roughness of the substrate. Previous studies on the pedicel have shown that it is used for attaching to smooth surfaces of the carrier (Faasch 1967; Mertins and Hartdegen 2003; Bajerlein and Błoszyk 2004). The influence of topography of the carrier body on attachment in phoretic Uropodina was discussed in detail by Bajerlein et al. (2013). *A. prodromus* has elytra with regularly alternating rows of fine punctures and flat interrows. Our observations have shown that most deutonymphs attached to elytra have the pedicellar disk fixed to the flat surface between pit rows (Fig. 2b, c). This confirms that not only surfaces covered by setae or bristles but also sculptured surfaces with microridges are avoided; instead, flat surfaces without sculpture are preferred. On the other hand, topical specificity in Uropodina cannot simply be explained by the topography of the surface, since in most cases deutonymphs occupied the rear parts of elytra, although the topography of different parts of elytra is quite similar.

Our results have revealed that the length of the pedicel within a species may be associated with the site of attachment. However, we did not find differences in pedicel length collected from *A. prodromus* and *A. distinctus*, although differences in body size between these beetles were statistically significant. This is in accordance with ecomorphological studies on attachment structures in parasitic associations, e.g., between monogeneans and fishes, and between feather lice and birds (Bush at al. 2006; Vignon et al. 2011). Even in the case of highly parasitic associations, attachment did not constrain host specificity. The pedicel enables phoretic deutonymphs to use a wide variety of hosts on the one hand, and, on the other hand, due to its chemical and physical properties, allows modification of its length. In this way, phoretic deutonymphs can adjust to the prevailing dispersal conditions and increase the probability of successful dispersal.

Our study showed that the density of phoretic deutonymphs on the carrier affects pedicel length. The mean length of pedicels increases with increasing mite density and, consequently, longer pedicels allow more deutonymphs to disperse. In our opinion, this is caused by decreasing surface availability, making the attachment of additional deutonymphs difficult. We have shown that the surface of a phoretic deutonymph is only about two times smaller compared to the area of the femur and trochanter in *A. distinctus* and only two and a half times smaller if compared with the same area in *A. prodromus*. This means that if phoretic deutonymphs of *U. orbicularis* would produce very short pedicels such as, e.g., deutonymphs of *Uroobovella pulchella* (Berlese, 1904) (Bajerlein et al. 2013), only ca. two individuals would be able to attach. *U. pulchella* is the smallest Uropodina species studied by us; phoretic deutonymphs of this species form an extremely short pedicel (ca. 13 μm) and appear as if they were placed directly on the carrier. The formation of a longer pedicel maintains the deutonymph at a certain distance from the attachment site and enables more deutonymphs to become attached. Moreover, the variable length of pedicels allows many deutonymphs to be distributed in three-dimensional space instead of one plane of the carrier surface. In our study, we often observed four, five, or more deutonymphs attached to the femur and a few of them produced pedicels of standard length (i.e., the length of the pedicel when only one deutonymph is attached) and one or two deutonymphs had longer pedicels. This suggests that pedicel length may reflect the sequence of deutonymph attachment: deutonymphs with shorter pedicels attach earlier, whereas deutonymphs with longer pedicels attach subsequently.

A commonly recorded phenomenon in phoresy of *U. orbicularis* is that deutonymphs travel in groups, and frequently, one deutonymph is attached next to the other, even if other beetle body parts are free of mites. As previously hypothesized (Bajerlein et al. 2013), dispersal in groups of immature stages may increase the chance to find a partner to mate after reaching the new habitat. Such intraspecific aggregation, known as the aggregation model of coexistence, has been studied for many ectoparasites and facilitates their coexistence (Morand et al. 1999; Simková et al. 2000; Presley 2011). Observations on phoresy made by Faasch (1967) have shown that phoretic deutonymphs prefer places already infested by deutonymphs. Moreover, Faasch (1967) noticed that even pedicels without deutonymphs stimulate attachment of additional deutonymphs. Our earlier observations on the localization of *U. orbicularis* on beetles (Bajerlein and Błoszyk 2004) have shown that phoretic deutonymphs are characterized by topical specificity. Attaching to the proper site has a priority even if it is already infested by other phoretic deutonymphs. Thus, two factors should be accounted when the phenomenon of group formation in *U. orbicularis* is explained: (1) the presence of previously attached mites of the same species, increasing the probability of finding a mating partner after reaching the new habitat, and (2) selection of a site that will provide safe dispersal.

As we confirmed in this study, phoretic deutonymphs are able to produce pedicels of varying length according to localization on the host and deutonymph density, which is related to the availability of area for attachment. However, the pedicel is a structure that not only enables phoretic dispersal but also the ability to control its length allows an increase in number of carried deutonymphs, increasing the chance of successful colonization of and mating in a new habitat. It should be emphasized that the costs of longer and shorter pedicel formation seems to be comparable, since pedicel length and diameter are inversely proportional (Bajerlein et al. 2013). This means that the diameter decreases as the length increases due to the expansion of newly formed pedicel without additional secretion of pedicellar substance.

Although mushroom-shaped adhesive organs have been the subject of many biomechanical studies, none of them involved termini of the pedicel in phoretic Uropodina. In fact, up to now, the pedicel has been described only morphologically and ecologically. Therefore, investigations on its biomechanical properties are needed to better understand the mechanism of phoretic deutonymph attachment to the carrier. Moreover, biomechanical and biochemical approaches are desirable, since many biomaterials known from arthropods may be of medical importance (Kuhbier et al. 2011; Kundu et al. 2013).
